# The ACSS2-PPARD-BCAT1 axis synchronously regulates branched-chain amino acid metabolism and development in pancreatic cancer: ACSS2 regulates BCAA metabolism and development in pancreatic cancer

**DOI:** 10.3724/abbs.2025111

**Published:** 2025-09-05

**Authors:** Haidi Chen, Zeng Ye, Lijun Liu, Wenyan Xu, Yihua Shi, Shunrong Ji, Xiaowu Xu, Xianjun Yu, Sen Hou, Yi Qin, Chenjie Zhou

**Affiliations:** 1 Department of Pancreatic Surgery Fudan University Shanghai Cancer Center Shanghai 200032 China; 2 Department of Oncology Shanghai Medical College Fudan University Shanghai 200032 China; 3 Shanghai Pancreatic Cancer Institute Shanghai 200032 China; 4 Pancreatic Cancer Institute Fudan University Shanghai 200032 China; 5 Department of Hepatobiliary and Pancreatic Surgery Xuchang Central Hospital Xuchang 461000 China

**Keywords:** pancreatic cancer, ACSS2, PPARD, BCAT1, acetylation, BCAA

## Abstract

The characteristics of the tumor microenvironment (TME) of pancreatic cancer include an abundant stroma, hypoxia, insufficient blood supply and high degree of immunosuppression. Therefore, overcoming the TME conditions to reach a hypermetabolic state is a concern for the treatment of pancreatic cancer. Previous studies have demonstrated that tumor cells adapt to the TME by activating or increasing the expression level of ACSS2 under metabolic stress. Our study focuses mainly on the relationship between ACSS2 and amino acid metabolism. We find that ACSS2 is generally highly expressed and promotes the proliferation and invasiveness of pancreatic cancer. Knockout of *ACSS2* reduces the catabolism of branched chain amino acids (BCAAs) by inhibiting the transcription of *BCAT1*. ACSS2 participates in the regulation of histone and transcription factor acetylation. Mechanistically, ACSS2 promotes acetylation at the H3K27 site in the *PPARD* promoter region to increase the transcription of *BCAT1* and ultimately alters the metabolic status of BCAAs. Moreover, the proliferation and invasion status induced by ACSS2 can be partly reversed by BCAT1 in pancreatic cancer cells. In summary, we believe that targeting the ACSS2-PPARD-BCAT1 axis has certain clinical value and can provide a new therapeutic strategy for the comprehensive treatment of pancreatic cancer.

## Introduction

Pancreatic cancer is a highly malignant tumor of the digestive system characterized by its insidious onset, difficulty in early diagnosis, and high propensity for invasion and metastasis. Most patients miss the opportunity for curative surgical resection by the time they seek medical attention. Even with curative surgical resection, recurrence and metastasis are common post-surgery [ 1, 2]. Additionally, pancreatic cancer is relatively insensitive to chemotherapy and tends to develop drug resistance [ 3 , 4]. Consequently, the overall 5-year survival rate for patients with pancreatic cancer is only 11% [5]. The incidence and mortality rates of pancreatic cancer are steadily increasing worldwide. In 2023, pancreatic cancer was the third leading cause of cancer-related death, and it is projected to become the second leading cause by 2040 [6]. Therefore, there is an urgent need to explore the molecular mechanisms underlying highly malignant biological features, identify specific therapeutic targets and optimize treatment strategies for pancreatic cancer.

Compared with the surrounding nonneoplastic pancreatic tissue, pancreatic cancer is distinguished by the presence of abundant stroma, leading to a tumor microenvironment (TME) characterized by hypoxia, acidity, and nutrient deficiency [ 7, 8]. To counteract the metabolic challenges posed by the TME, these tumor cells have evolved various strategies, including the enhancement of lipogenesis and the acquisition of nutrients. Acetate is the main carbon source for energy metabolism and is converted to acetyl-CoA by acetyl-CoA synthetase, thereby affecting cellular metabolism, lipogenesis and functional regulation mediated by protein acetylation [ 9, 10]. Acyl-coenzyme A synthetase short-chain family member 2 (ACSS2) can help tumors adapt to the TME through metabolic reprogramming [ 11, 12]. Studies have revealed that branched-chain amino acids (BCAAs), including valine, leucine and isoleucine, affect the tricarboxylic acid (TCA) cycle and lipid metabolism and play important roles in tumors [13]. Branched-chain amino acid transaminase (BCAT) reversibly promotes the first step of BCAA catabolism, producing branched-chain α-ketoacids (BCKAs). There are two subtypes of BCAT, BCAT1 and BCAT2, which are located in the cytoplasm and mitochondria, respectively [ 14, 15]. BCAAs are important nitrogen sources for tumor cells, and the associated metabolic enzyme levels are elevated and play a role in promoting proliferation in pancreatic cancer [ 16 , 17]. However, the relationship between ACSS2 and BCAA metabolism is still unclear. Therefore, we elucidated the relationship between these two factors and identified key aspects. These findings are expected to provide an ideal strategy for the treatment of pancreatic cancer.

In the present study, we investigated the effect of ACSS2 on BCAA metabolism. We found that ACSS2 promoted the catabolism of BCAAs. Mechanistically, ACSS2 further promotes the transcription of *BCAT1* by affecting H3K27ac in the *PPARD* promoter region and ultimately affecting BCAA metabolism. Moreover, BCAT1 reversed the effects of ACSS2 on the proliferation and invasion of pancreatic cancer cells.

## Materials and Methods

### Cell culture

Human-derived pancreatic cancer cell lines, including HPAF-II, PANC-1, BxPC-3, AsPC-1, Capan-1, SW1990 and MIA PaCa-2, and a pancreatic cell line (H6C7) were obtained from the American Type Culture Collection (ATCC; Manassas, USA), subjected to authentication via DNA fingerprinting in 2016 and subsequently passaged in our laboratory within a six-month period post-receipt. The PANC-1, H6C7 and Capan-1 cell lines were cultured in DMEM (Gibco, Carlsbad, USA) supplemented with 10% FBS (Gibco). The SW1990 cell line was cultured in L-15 medium (Gibco) supplemented with 10% FBS. HPAF-II cells were cultured in MEM (Gibco) supplemented with 10% FBS and 1% NEAA (Gibco). MIA PaCa-2 cells were cultured in DMEM (Gibco) supplemented with 10% FBS and 2.5% horse serum (Gibco). The BxPC-3 and AsPC-1 cell lines were cultured in RPMI medium (Gibco) supplemented with 10% FBS. Streptomycin (100 μg/mL) and penicillin (100 U/mL) were added to all culture media to ensure sterility and health.

### Chemicals

ERG240 (BCAT1 inhibitor, HY-W193545A, 10 μg/mL) and Ac-CoA synthase inhibitor 1 (ACSS2 inhibitor, HY-104032, 5 μM) were purchased from MedChemExpress (Monmouth Junction, USA) [ 18, 19]. Acetate (S8750, 1 mM) was purchased from Sigma-Aldrich (St Louis, USA) [19] .

### Cell viability and colony formation assay

Cell viability was assessed as previously described [20]. A total of 3000 cells were seeded in 96-well plates, and the CCK-8 working solution was obtained by mixing 10% CCK-8 reagent (C0038; Beyotime, Shanghai, China) with 90% serum-free medium. Finally, after 48–72 h, the medium was replaced by CCK-8 working solution, and the cells were incubated in an incubator (37°C for 2 h) for inspection (wavelength of 450 nm).

In brief, the colony formation assay was performed as follows. In six-well plates, 600 cells were seeded in each well and cultured under appropriate conditions (37°C) for 2 weeks, followed by fixation, staining, and quantification.

### Plasmids

Flag-tagged ACSS2 and flag-tagged BCAT1 were cloned and inserted into a pCDH-CMV-MCS-EF vector (System Biosciences, San Diego, USA) to generate ACSS2 and BCAT1 expression plasmids, respectively. siRNA duplexes against *PPARD* (M-003584-02-0010; Dharmacon, Lafayette, USA) were transfected into pancreatic cancer cells using Lipofectamine 3000 (Invitrogen, Carlsbad, USA) [21].

### CRISPR/Cas9 knockout

CRISPR/Cas9 knockout was carried out as previously described [22]. The two ACSS2-specific gRNA sequences were 5′-CTGGCTATGGTACCACCGGG-3′ and 5′-GGCCTGGCTATGGTACCACC-3′.

### RNA isolation and quantitative real-time PCR

RNA isolation and quantitative real-time PCR (qRT-PCR) was carried out as previously described [ 23– 25 ]. In brief, cDNA was obtained by using TRIzol reagent (Invitrogen) and a PrimeScript RT Reagent kit (TaKaRa, Dalian, China). An ABI 7900HT real-time PCR system (Applied Biosystems, Foster City, USA) was used to determine the expression levels of *ACSS2*, *PPARD* and *BCAT1*. The primers used were as follows: human *ACSS2*: 5′-AAAGGAGCAACTACCAACATCTG-3′ (forward), 5′-GCTGAACTGACACACTTGGAC-3′ (reverse); human *PPARD* (Thermo Fisher Scientific, Waltham, USA; Hs00987011_m1); human BCAT1: 5′-ACCTGTCCGAGACCTATGGG-3′ (forward), 5′-CCAGGCTCTTACATACTTGGGA-3′ (reverse); and human *ACTB*: 5′-CTGGAACGGTGAAGGTGACA-3′ (forward), 5′-AAGGGACTTCCTGTAACAACGCA-3′ (reverse).

### Western blot analysis

Western blot analysis was carried out as previously described [20]. Anti-ACSS2 (ab66038), anti-PPARD (ab178866), anti-VIM/vimentin (ab8978) and anti-SNAI1 (ab216347) antibodies were purchased from Abcam (Cambridge, UK). Anti-CDH1/E-cadherin (3195) and anti-CDH2/N-cadherin (13116) antibodies were purchased from Cell Signaling Technology (Danvers, USA). Anti-BCAT1 (13640-1-AP) and anti-ACTB (60008-1-lg) antibodies were purchased from Proteintech (Chicago, USA).

### Wound healing assay and Transwell migration and invasion assay

Five horizontal lines were marked on the back of each well in 6-well plates at 0.5–1 cm intervals. One million cells were seeded and cultured at 37°C under 5% CO _2_. After 24 h, a vertical line was drawn through the wells at 100% confluence using a 200-μL pipette tip. The cells were then washed with PBS and treated with serum-free medium containing the test chemicals. After another 24 h, the cells were imaged under a microscope (Vert.A1; ZEISS, Wetzlar, Germany) and analyzed semiquantitatively using ImageJ software.

The specific steps of the Transwell migration and invasion assay were as follows. In 20-well plates, 800 μL of complete culture medium was added to each well. To the upper chamber, 200 μL of serum-free medium containing 6 × 10 ^4^ cells and the appropriate chemicals, including or excluding Matrigel, were added. Following a 24-h incubation period, the cells in the lower chamber were subjected to washing, fixation, and staining procedures. Subsequently, random fields were selected for the quantification of the number of cells.

### Chromatin immunoprecipitation assay

An EZ-ChIP Kit (Millipore, Billerica, USA) was used to perform chromatin immunoprecipitation (ChIP) assays as previously described [20]. The sequences of the primers used to test *BCAT1* promoter occupancy were as follows: 5′-TGATGACGGAATGGCAAT-3′ (forward) and 5′-CTCACTCACTGCTCACTC-3′ (reverse). The sequences of the primers used to test the level of H3K27ac in the *PPARD* promoter region were as follows: 5′-CAGTCCGAGACGAGAGAAG-3′ (forward) and 5′-CTACATGGCAACGCACGA-3′ (reverse).

### Promoter activity assessment by dual-luciferase assay

The promoter region of *BCAT1*, corresponding to positions –2000 to +250 from the transcription start site, was inserted into the pGL3-Basic vector. Luciferase assays were conducted in accordance with the manufacturer’s guidelines for the assay kits (Promega, Madison, USA).

### PDAC patient-derived organoid culture and drug test assays

Healthy patient-derived organoids (PODs) were passaged and seeded into 48-well plates (3548l, Costar, Washington, USA) at an adjusted density. Each well contained approximately 40 ± 10 organoids, 5 μL of Matrigel and 300 μL of culture medium (OrganoPro ^TM^ Tumour Organoid Culture kit; K2 ONCOLOGY, Beijing, China). The PDO size, with a target diameter of 100 mm, was used to determine the optimal time for *in vitro* treatment. For drug testing, the organoid culture medium was replaced by 300 μL of drug-containing medium, which was changed every two days. PDOs were photographed daily for 6 days posttreatment under a microscope (Vert.A1; ZEISS). The size of viable PDOs was evaluated via Image-Pro Plus 6.0 software (Media Cybernetics, Inc., Bethesda, USA). On the sixth day posttreatment, PDO viability was assessed using the Cell-Titer-Glo ^®^ 3D Cell Viability Assay kit (G9683; Promega).

### Immunohistochemical staining

From 2012 to 2014, the clinical tissue samples were clinically and histopathologically diagnosed at Fudan University Shanghai Cancer Center (FUSCC). This study was approved by the Institutional Research Ethics Committee of Fudan University Shanghai Cancer Centre (ethical code: 050432-4-1212B), and written informed consent was obtained from all patients. Immunohistochemical (IHC) staining was conducted according to previously established methods [20]. Anti-ACSS2 (1:100; Abcam) and anti-BCAT1 (1:50; Proteintech) antibodies were used to assess protein expression levels.

### ELISAs and RNA-seq

BCKA levels were measured using a human ELISA kit (YJ912536; mlbio, Seoul, South Korea) according to the manufacturer’s instructions. RNA-seq was performed as described previously (GSE279400) [23] .

### Statistical analysis

The experiments were conducted with a minimum of three replicates to ensure the reliability of the results. Data analysis was performed using SPSS version 26.0 software or GraphPad Prism version 9. To compare the differences between two groups, two-tailed unpaired Student’s *t* tests were used. Correlation analysis was employed through Spearman correlation. Survival curves were generated using the Kaplan-Meier method, and differences were assessed using the log-rank test. Statistical significance was determined when *P* < 0.05.

## Results

### High ACSS2 expression is associated with poor prognosis in patients with PDAC

**Figure 1:**
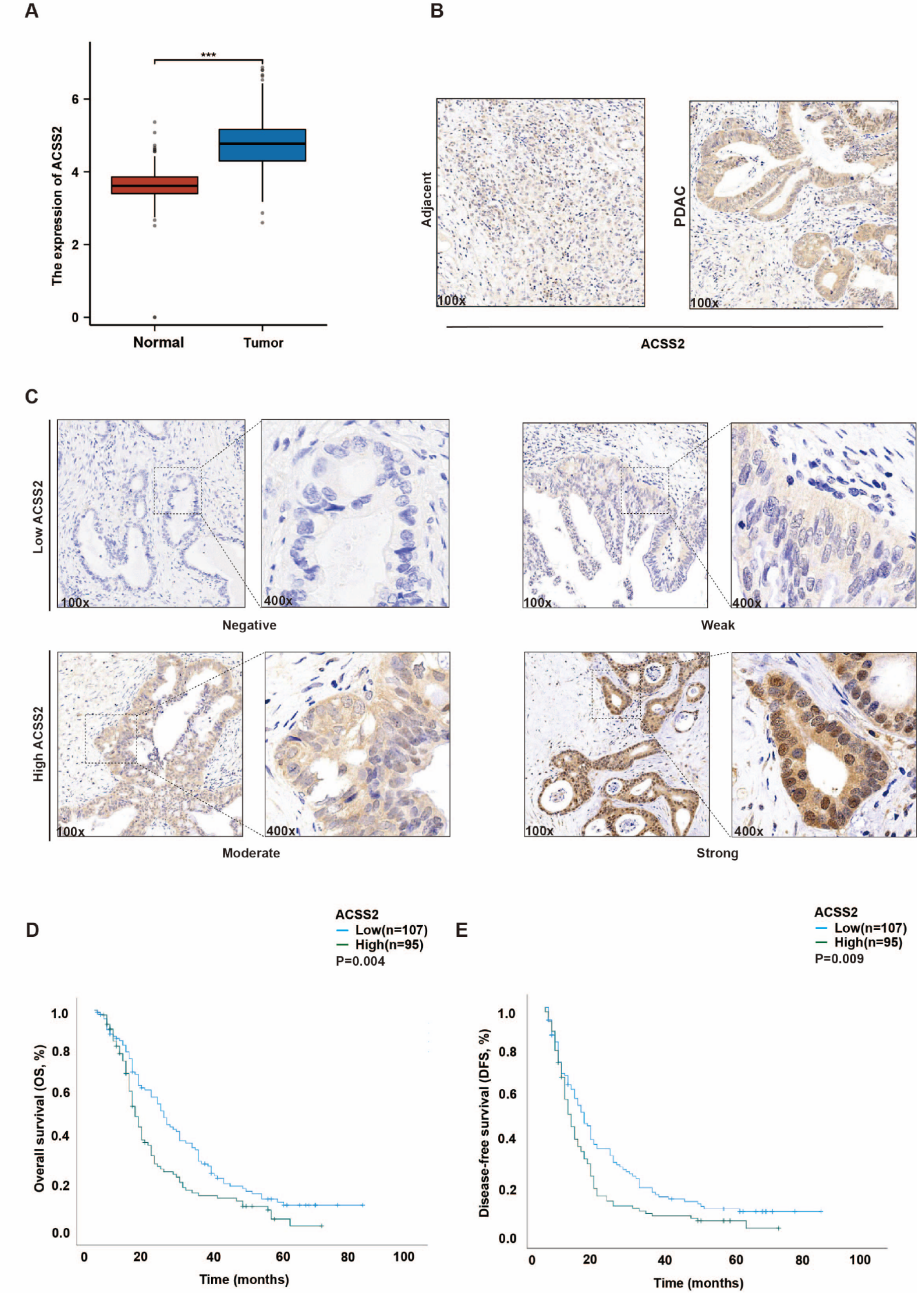
Figure 1 High ACSS2 expression is associated with poor prognosis in patients with PDAC (A) In the GEPIA dataset, pancreatic cancer tissues presented dramatically higher levels of ACSS2 transcription than normal tissues did (num (T) = 179, num (P) = 171, *** P < 0.001 as determined by t test). (B) Images of IHC staining for ACSS2 are shown for PDAC and adjacent normal tissues (original magnification, ×100). (C) The proportion and intensity of ACSS2 expression in PDAC patients were assessed by IHC staining (original magnification, ×100; inset original magnification, ×400). (D,E) OS (n = 202, P = 0.004, as determined by the log-rank test) and DFS (n = 202, P = 0.009, as determined by the log-rank test) were evaluated by Kaplan-Meier analysis to determine the values for ACSS2.

The Gene Expression Profiling Interactive Analysis dataset revealed that ACSS2 transcription was significantly greater in pancreatic cancer tissues than in normal tissues ( Figure 1A). To evaluate the protein level of ACSS2, we performed IHC staining of the tumor microarray in the FUSCC cohort with 202 patient samples, and ACSS2 protein expression showed the same trend ( Figure 1B). We ranked the proportion score as 0 (0%–5%), 1 (6%–25%), 2 (26%–50%), 3 (51%–75%) or 4 (76%–100%), defined the intensity score as 0 (no colouration), 1 (pale yellow), 2 (clay bank) or 3 (brown) and determined the IHC score as the proportion score multiplied by the intensity score. Subsequently, according to the IHC score of ACSS2 expression, we divided the cohort into a high-expression group (IHC score ≥ 6) and a low-expression group (IHC score < 6) ( Figure 1C). Patients with low ACSS2 expression had longer OS ( *P* = 0.004, median time: 15 months) and DFS ( *P* = 0.009, median time: 9 months) ( Figure 1D,E). Therefore, we believe that the use of *ACSS2* as an oncogene can affect the prognosis of patients with pancreatic cancer.

### ACSS2 is essential for the proliferation and invasion in PDAC

**Figure 2:**
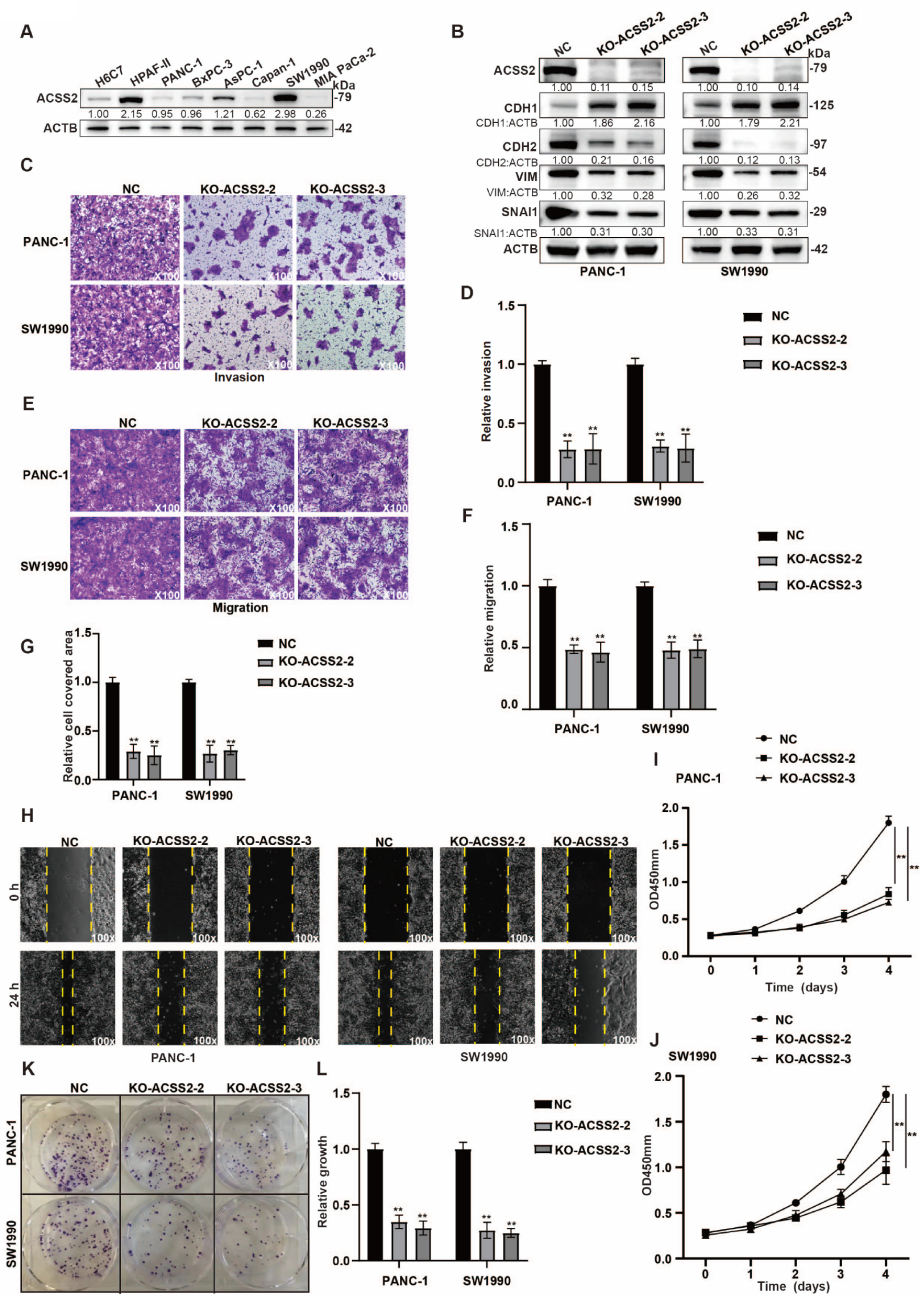
Figure 2 ACSS2 is essential for the proliferation and invasiveness of PDAC (A) Western blot analysis of ACSS2 expression was performed in seven pancreatic cancer cell lines and the H6C7 cell line. (B) The effects of ACSS2 expression on EMT markers (CDH1, CDH2, VIM and SNAI1) in PANC-1 and SW1990 cells were examined by western blot analysis. (C–F) Invasiveness and migration induced by ACSS2 expression were assessed by a Transwell assay (t test, **P < 0.01) (original magnification, ×100). (G,H) Cell motility was evaluated by a wound healing assay in PANC-1 and SW1990 cell lines with or without ACSS2 expression (t test, **P < 0.01) (original magnification, ×100). (I,J) CCK-8 assay was performed to detect proliferation in PANC-1 and SW1990 cell lines with or without ACSS2 expression (t test, **P < 0.01). (K,L) The influence of ACSS2 silencing was tested by colony formation assay and quantified in pancreatic cancer cell lines (t test, **P < 0.01) (original magnification, ×100). ImageJ software was used to quantify the grayscale value of each western blot band.

Owing to the increased expression of ACSS2 in tumor tissues and its influence on prognosis, we further clarified the role of ACSS2 in the malignant biological behavior of PDAC. We first detected ACSS2 protein expression in a pancreatic cell line (H6C7) and seven pancreatic cancer cell lines (HPAF-II, PANC-1, BxPC-3, AsPC-1, Capan-1, SW1990 and MIA PaCa-2) and discovered that, compared with pancreatic cells, pancreatic cancer cell lines always presented high *ACSS2* expression ( Figure 2A). Stable PANC-1 and SW1990 cell lines with *ACSS2* knockout were constructed on the basis of comparisons with pancreatic cancer cell lines, and the effects of the knockout were verified ( Figure 2B). The results of the western blot analysis revealed that the loss of ACSS2 expression reduced the induction of epithelial-mesenchymal transition (EMT) ( Figure 2B). We evaluated the function of ACSS2 in motility through Transwell assays and found that insufficient expression of ACSS2 decreased migration and invasion ( Figure 2C–F). Consistent with these results, the results of the Transwell assays revealed that *ACSS2* silencing delayed the degree of wound closure ( Figure 2G,H). Therefore, *ACSS2*-knockout pancreatic cancer cells noticeably inhibited cell proliferation and colony formation ability ( Figure 2I–L). These results suggest that ACSS2 is essential for the proliferation and invasion in PDAC.

### ACSS2 increases BCAA metabolism by upregulating BCAT1

**Figure 3:**
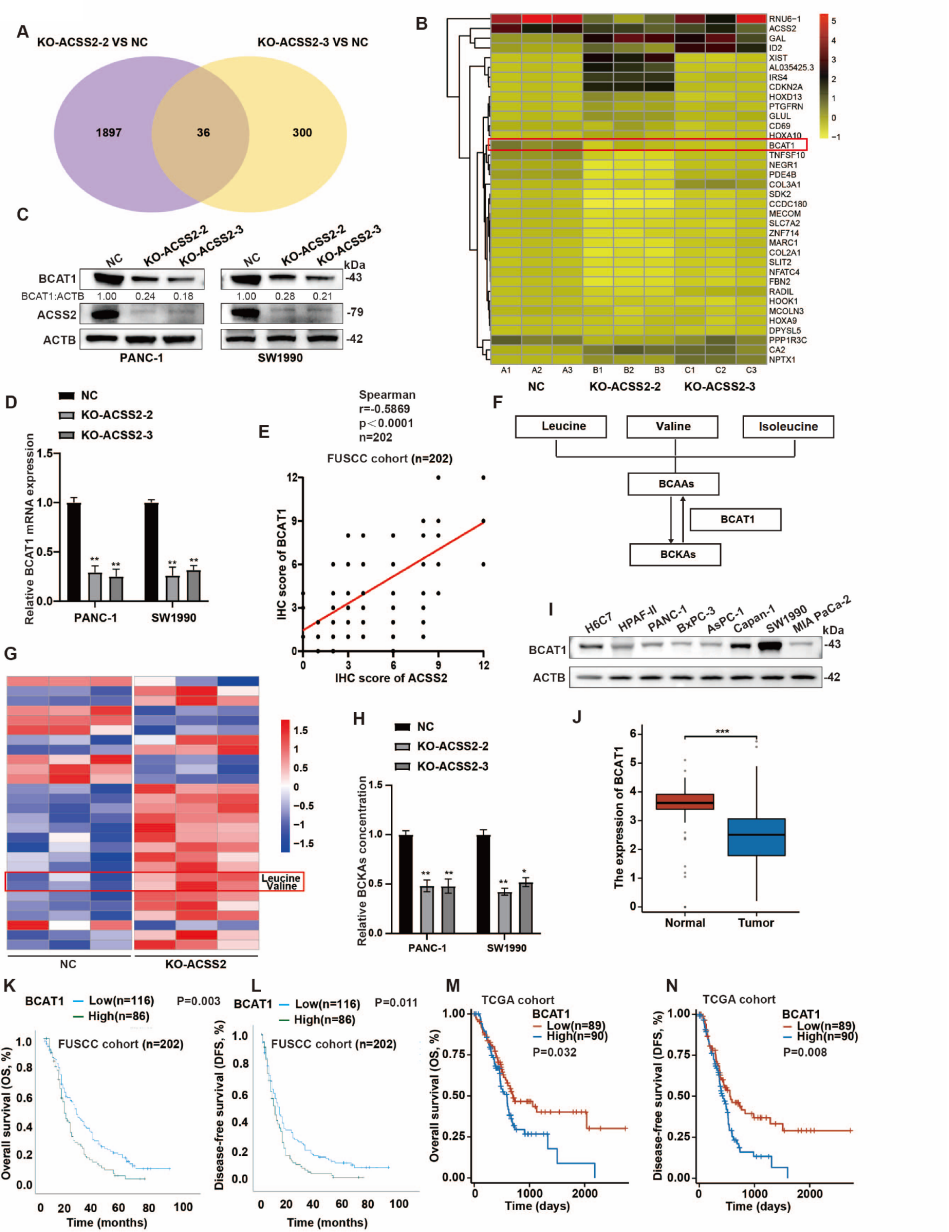
Figure 3 ACSS2 increases BCAA metabolism by upregulating BCAT1 (A,B) RNA-seq was conducted to detect significantly differentially expressed genes related to ACSS2 in both ACSS2 knockout groups (n = 3). (C,D) Western blot analysis and qRT-PCR were performed to confirm the relationship between ACSS2 and BCAT1 (t test, **P < 0.01). (E) Correlation analysis of the IHC staining score between ACSS2 and BCAT1 was performed in the FUSCC cohort (n = 202, Spearman’s r = 0.5869, P = 0.0001). (F) Pattern diagram revealing that BCAT1 is one of the key enzymes involved in the first step of the reversible catabolism of BCAAs. (G) Amino acid metabolism analysis was conducted to investigate the influence of ACSS2 knockout (n = 3). (H) The concentration of BCKAs, which are metabolites of BCAA, was tested by ELISA in the PANC-1 and SW1990 cell lines with or without ACSS2 expression ( t test, *P < 0.05, ** P < 0.01). (I,J) In seven pancreatic cancer cell lines, H6C7 cells and the GEPIA dataset, the level of ACSS2 expression was assessed [num (T) = 179, num (P) = 171, ***P < 0.001 as determined by t test]. (K,L) OS (n = 202, P = 0.003) and DFS ( n = 202, P = 0.011) were identified by Kaplan-Meier analysis to reveal the effect of BCAT1 in the FUSCC cohort (log-rank test). (M,N) OS (P = 0.032) and DFS (P = 0.008) were confirmed by Kaplan-Meier analysis to estimate the function of BCAT1 in the TCGA cohort (log-rank test).

ACSS2-mediated acetylation regulation and substance metabolism are associated with tumorigenesis [26]. To explore the potential molecular mechanism by which ACSS2 regulates tumor development, we conducted RNA sequencing analysis to identify genes that were significantly differentially expressed between the control and *ACSS2* knockout groups ( Figure 3A,B). Notably, BCAT1 was markedly downregulated in the *ACSS2* knockout groups compared with the control group. To further verify the regulatory relationship between ACSS2 and BCAT1, we conducted western blot analysis and qRT-PCR, which revealed that ACSS2 positively regulated BCAT1 at both the protein and mRNA levels ( Figure 3C,D). We also found the same trend in the correlation analysis of the IHC staining score between ACSS2 and BCAT1 in the FUSCC cohort ( Figure 3E). Conversely, BCAT1 promoted the first step of BCAA catabolism to produce branched-chain keto acids (BCKAs) ( Figure 3 F) [23]. Therefore, we speculated that ACSS2 could affect the metabolism of BCAA. To evaluate the changes in amino acid levels caused by ACSS2, we conducted a targeted metabolomic study and detected that BCAAs (leucine and valine) were prominently upregulated in the *ACSS2* knockout group ( Figure 3G). To clarify whether the consumed BCAAs were converted into BCKAs, an ELISA kit was used to determine the concentrations of BCKAs (metabolites of BCAAs). The results revealed that BCKAs were downregulated after *ACSS2* knockout ( Figure 3H). We conducted western blot analysis to evaluate the protein expression level of BCAT1 and found that BCAT1 expression was low in most pancreatic cancer cell lines ( Figure 3I). In the TCGA cohort, the analysis results suggested a higher expression level in normal tissues ( Figure 3J). On the basis of the difference in BCAT1 expression between tumor tissues and normal tissues, we generated Kaplan-Meier survival curves and performed log rank tests and found that patients with high BCAT1 expression had worse overall survival (OS) and disease-free survival (DFS) in both the TCGA cohort and the FUSCC cohort ( Figure 3K–N). Therefore, we believe that ACSS2 increases BCAA metabolism by upregulating BCAT1.

### ACSS2 enhances BCAT1 expression through PPARD

**Figure 4:**
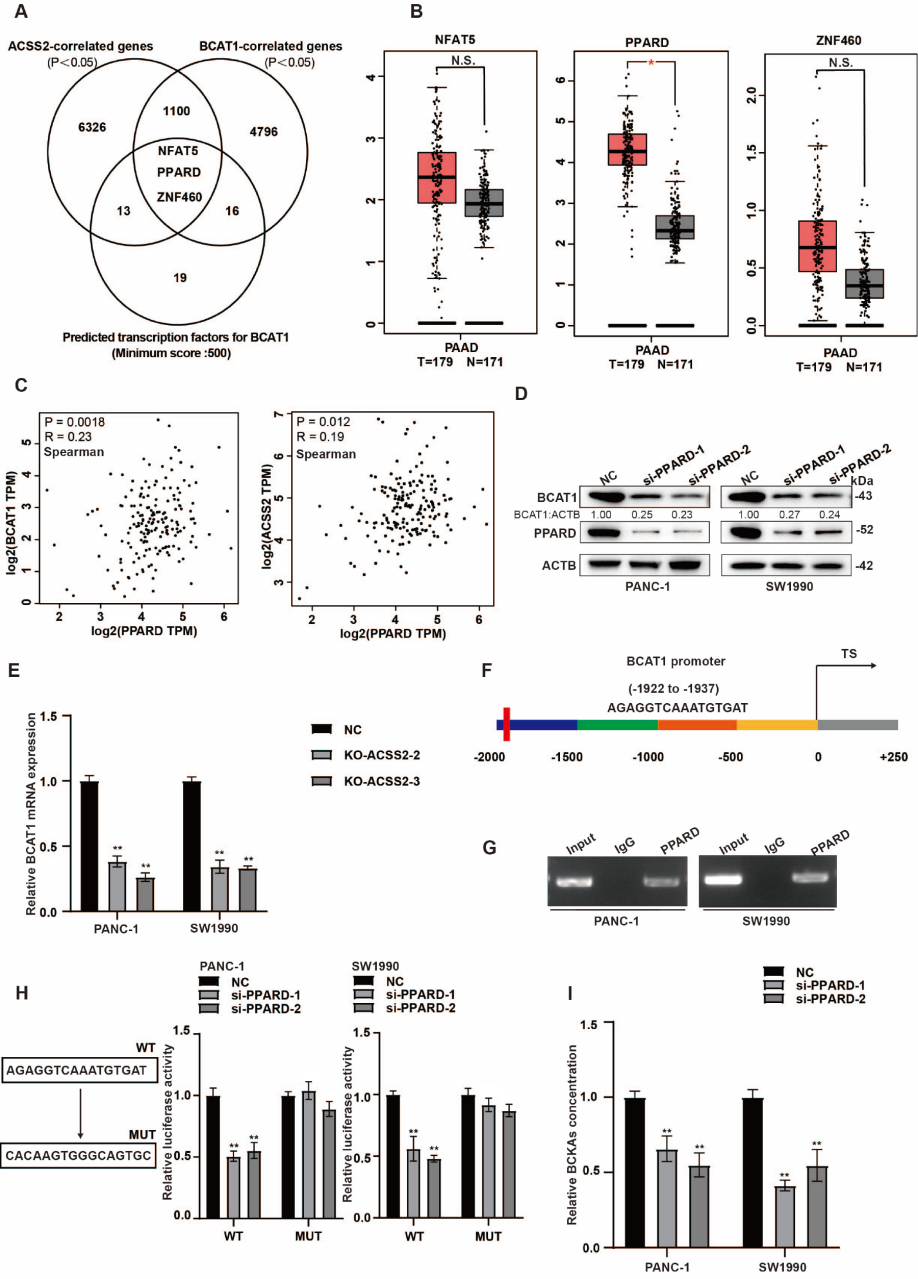
Figure 4 ACSS2 enhances BCAT1 expression through PPARD (A) Venn diagram revealing that the transcription factors associated with BCAT1 were predicted by a public database. (B) In the GEPIA dataset, only PPARD, one of three potential transcription factors of BCAT1, had a significantly higher level of expression in pancreatic cancer tissues than in normal tissues [num (T) = 179, num (P) = 171, *P < 0.05, t test]. (C) A correlation between ACSS2 and PPARD or BCAT1 expression was identified in the TCGA cohort. (D,E) PPARD silencing reduced BCAT1 protein and mRNA expression levels (t test, **P < 0.01). (F,G) A ChIP assay was performed with an anti-PPARD antibody to confirm the potential binding site of PPARD on the BCAT1 promoter. (H) A dual-luciferase assay was conducted to assess the effect of PPARD (WT and MUT binding sites) on the promoter activity of BCAT1. **P < 0.01. (I) The concentration of BCKAs in the PANC-1 and SW1990 cell lines treated with si-PPARD or NC was determined by ELISA. **P < 0.01.

ACSS2 not only plays a crucial role in material and energy metabolism but also participates in the regulation of various acetylation processes, such as histone and transcription factor acetylation [26]. Accordingly, we speculated that ACSS2 regulates BCAT1 by affecting a transcription factor. To predict potential transcription factors mediating ACSS2-upregulated BCAT1 expression, we analyzed pancreatic cancer tissue samples from the TCGA database and identified 7442 ACSS2-related genes ( *P* < 0.05) and 5915 BCAT1-related genes ( *P* < 0.05) ( Figure 4A). According to the analysis results of the JASPAR database and the UCSC Genome Browser (minimum score: 500), 51 potential transcription factors were found for BCAT1. Next, we examined the intersection of these three gene clusters and identified three potential transcription factors for BCAT1 (NFAT5, PPARD and ZNF460) ( Figure 4A). Among these transcription factors, only PPARD was highly expressed in tumor tissues ( *P* < 0.05), whereas NFAT5 and ZNF460 were not significantly differentially expressed ( Figure 4B). PPARD primarily plays a tumorigenic role in pancreatic cancer, promoting tumor progression by regulating proliferation, metabolism, inflammation, and metastasis, among other pathways. Although it holds potential as a therapeutic target, further exploration of its complex biological mechanisms and clinical translation strategies are still needed [27]. PPARD was associated with ACSS2 ( *P* = 0.0018, R = 0.23) and BCAT1 ( *P* = 0.012, R = 0.19) expressions in patients’ pancreatic tumor tissues in the TCGA cohort ( Figure 4C). We found that *PPARD*-silenced pancreatic cancer cells presented lower levels of BCAT1 mRNA and protein ( Figure 4D,E). To verify the combination of the promoter regions of *BCAT1* and *PPARD*, we analyzed the promoter region of *BCAT1* and performed ChIP with an anti-PPARD antibody, and the results revealed that PPARD could bind to the promoter region of *BCAT1* ( Figure 4F,G). To determine whether PPARD can transcriptionally activate BCAT1, we conducted a dual‐luciferase assay and found that PPARD downregulation significantly decreased the luciferase activity of the WT pGL3-BCAT1 promoter but not the MUT pGL3-BCAT1 promoter ( Figure 4H). These results indicated that PPARD bound to the *BCAT1* promoter and increased *BCAT1* transcription. Therefore, we also tested the level of BCKAs and found that *PPARD* silencing indeed reduced the concentration of BCKAs ( Figure 4I). These results suggest that ACSS2 upregulates BCAT1 through PPARD.

### ACSS2 promotes PPARD expression via H3K27ac

**Figure 5:**
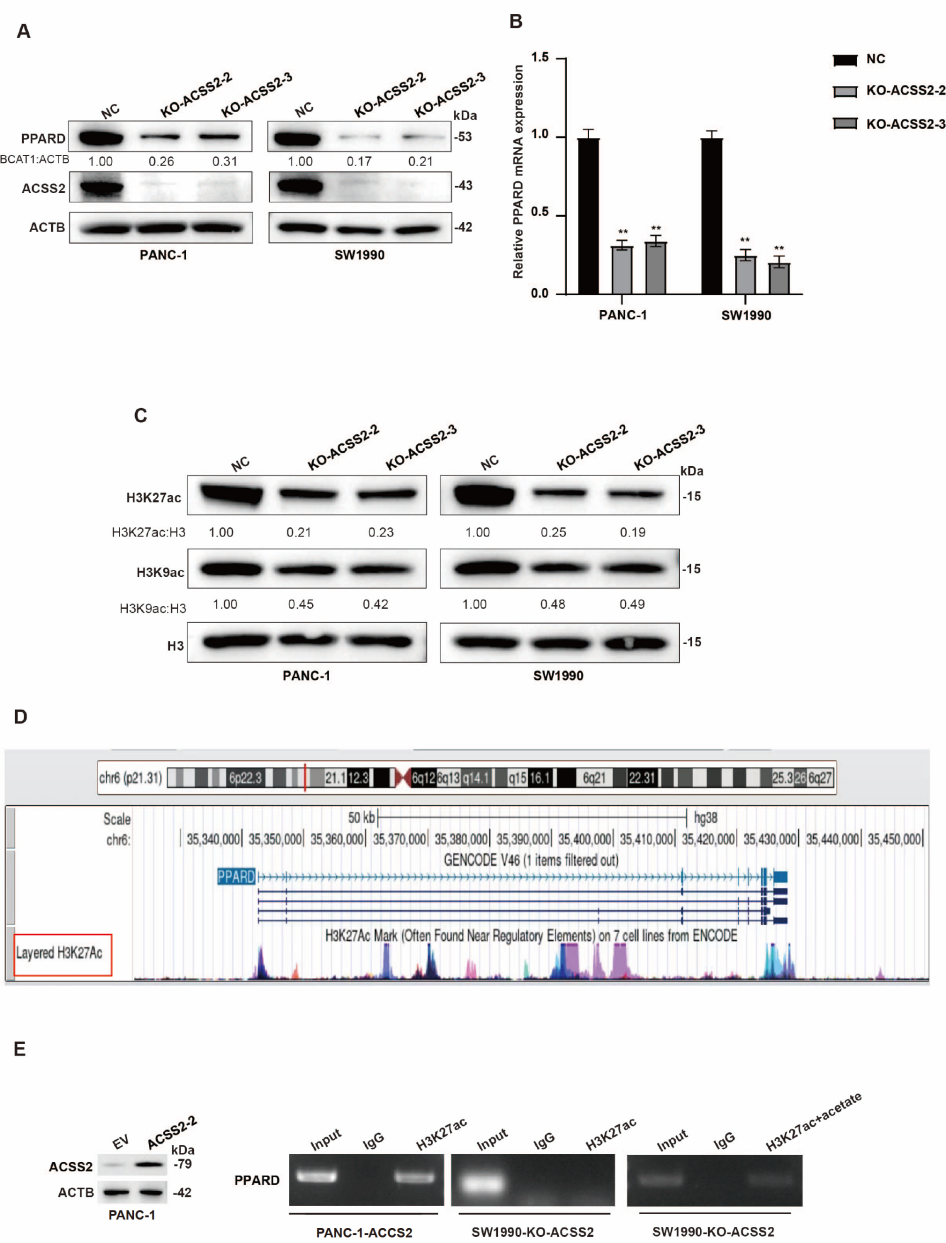
Figure 5 ACSS2 promotes PPARD expression via H3K27ac (A,B) The protein and mRNA expressions of PPARD were confirmed in PANC-1 and SW1990 cells with ACSS2 silencing. **P < 0.01. (C) Western blot analysis of the protein levels of H3K27ac and H3K9ac in PANC-1 and SW1990 cells with or without ACSS2 expression. (D) H3K27ac enrichment at the PPARD promoter was confirmed by UCSC. (E) A ChIP assay was used to test H3K27ac enrichment at the PPARD promoter in PANC-1 and SW1990 cells.

We performed western blot analysis and qRT-PCR and found that ACSS2 increased the expression of PPARD at the mRNA and protein levels ( Figure 5A,B). Research has shown that ACSS2 can regulate transcription factor acetylation to affect substance metabolism and tumorigenesis [26]. Moreover, Divya *et al*. [28] reported that ACSS2 could increase the expression of H3K27ac and H3K9ac in PDAC. Therefore, we hypothesized that ACSS2 might control H3K27ac and H3K9ac to affect the expression of PPARD. First, we conducted protein level validation, and the results revealed that the H3K27ac level was significantly lower than the H3K9ac level under *ACSS2* knockout conditions, which suggested that ACSS2 regulated PPARD mainly via H3K27ac ( Figure 5C). The *PPARD* promoter region was dramatically enriched with H3K27ac according to UCSC ( http://genome.ucsc.edu/) ( Figure 5D). To further determine the H3K27ac modification level in the promoter region of *PPARD*, we performed a ChIP-PCR assay and demonstrated that an increase in ACSS2 expression clearly increased the H3K27ac modification level in the promoter region of *PPARD*; however, a decrease in ACSS2 expression produced the opposite result ( Figure 5E). The addition of acetate could increase the acetylation of H3K27 in PDAC [28]. Our results also revealed that acetate treatment increased the H3K27ac modification level in the promoter region of *PPARD* ( Figure 5E). Therefore, we demonstrated that ACSS2 promoted PPARD expression via H3K27ac modification.

### BCAT1 alters the proliferation and invasion status induced by ACSS2 in PDAC

**Figure 6:**
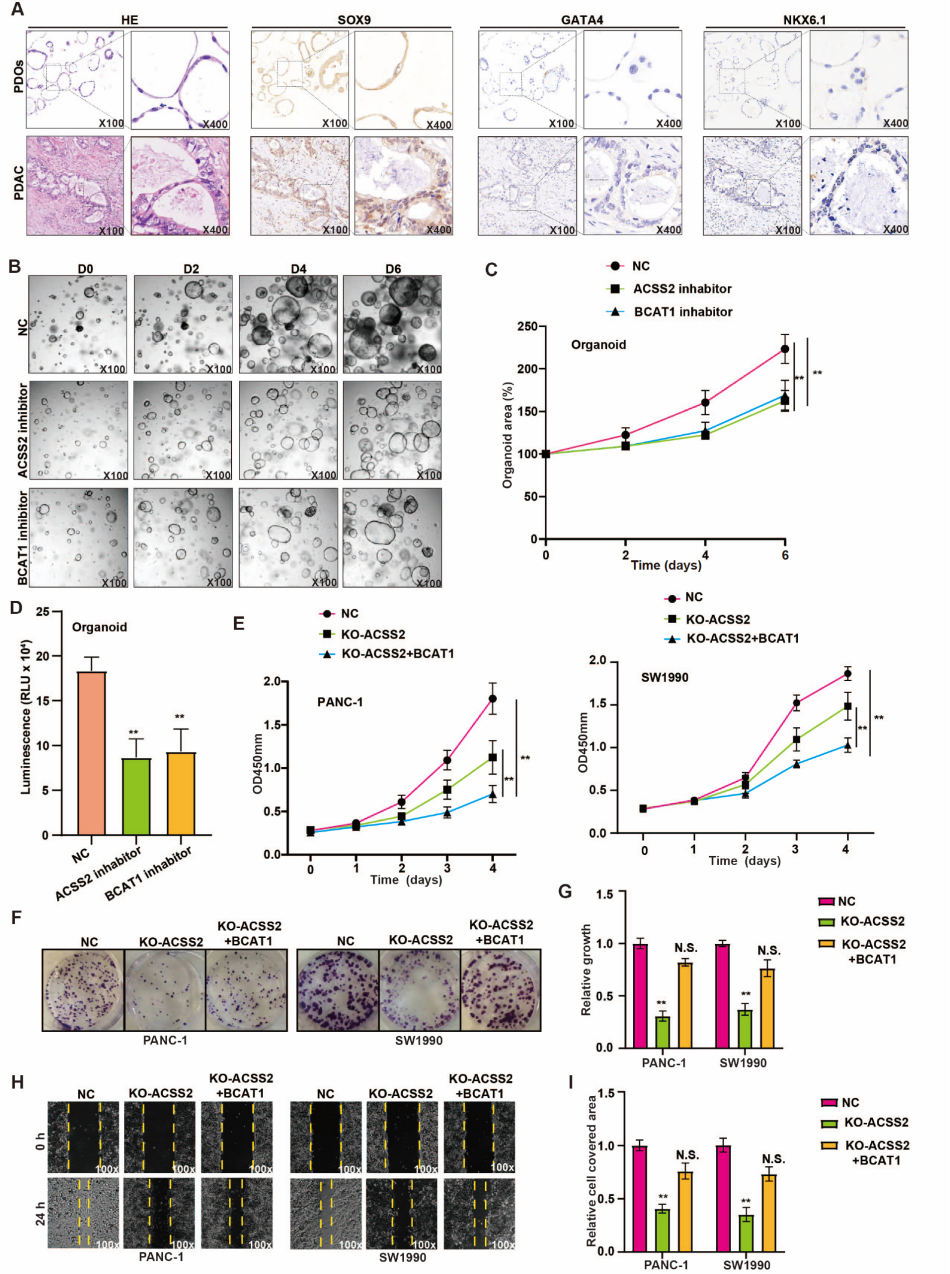
Figure 6 BCAT1 alters the proliferation and invasion status induced by ACSS2 in PDAC (A) The expression of HE, SOX9, GATA4 and NKX6.1 in PDAC patients and PDOs was evaluated via IHC staining (original magnification, ×100; inset original magnification, ×400). (B,C) PDO size (left) and area (right) were tested to evaluate the effects of the ACSS2 inhibitor and BCAT1 inhibitor (original magnification, ×100). (D) The viability of PDOs treated with the ACSS2 inhibitor or BCAT1 inhibitor was validated by the CellTiter-Glo 3D cell viability assay. (E) Proliferation was confirmed by CCK-8 assay in pancreatic cancer cell lines. (F–I) Wound healing and colony formation assays were conducted to assess the motility and proliferation of PANC-1 and SW1990 cells with or without ACSS2 expression. **P < 0.01.

To verify that the organoids originate from pancreatic tumors and maintain representative glandular tubular structures in pancreatic cancer patients, we conducted IHC staining, including HE, SOX9 (a marker of ductal differentiation in the pancreas), GATA4 (an acinar cell marker) and NKX6.1 (an islet cell marker) staining. We found that both PDOs and patient-derived pancreatic cancer tissues were positive for SOX9 but negative for GATA4 and NKX6.1, and had similar representative structures according to HE staining ( Figure 6A). We speculated that BCAT1, as an oncogene, might affect the proliferation and invasion caused by ACSS2 ( Figure 2I–L). PDOs were subsequently treated with the ACSS2 inhibitor or BCAT1 inhibitor and subjected to size analysis for one week. A CellTiter-Glo 3D cell viability assay was used to test the viability of PDOs on the seventh day. The experimental results suggested that, similar to the ACSS2 inhibitor, the BCAT1 inhibitor inhibited the growth and viability of PDOs ( Figure 6B–D). CCK-8 and colony formation assays were used to assess cell viability, and we found that BCAT1 overexpression partially alleviated the suppression of proliferation caused by reduced ACSS2 levels in pancreatic cancer cells ( Figure 6E,F). In addition, BCAT1 overexpression reversed the decrease in invasiveness caused by *ACSS2* silencing to some extent ( Figure 6H,I). In summary, BCAT1 shifted the proliferation and invasion status induced by ACSS2 in PDAC.

## Discussion

Pancreatic cancer is a highly malignant tumor. Owing to the difficulty in early detection and diagnosis, the window period for radical resection is very small, and it is very easy for patients to relapse after surgery [29]. In recent years, the increasing incidence of pancreatic cancer has led to a gradual increase in research on the treatment of pancreatic cancer. The characteristics of the tumor microenvironment of pancreatic cancer include a rich stroma, hypoxia, insufficient blood supply and high immunosuppression [30]. It is difficult to apply traditional chemoradiotherapy for pancreatic cancer [31]. However, pancreatic cancer is also a type of high-metabolism tumor, and metabolic reprogramming to overcome the lack of a tumor microenvironment has become a popular research topic. Tumor metabolic reprogramming is considered an emerging mechanism of therapeutic resistance, and it can be used to develop new strategies to treat pancreatic cancer [32].

Energy metabolism plays an important role in the development of pancreatic cancer. Among the various types of energy metabolism, lipid metabolism is involved in tumor cell metabolism reprogramming, which alleviates the insufficient supply of oxygen and nutrients and provides sufficient energy for the growth and proliferation of cancer cells [33]. ACSS2 bidirectionally regulates lipid metabolism [26]. Under normal conditions without damage or stress, ACSS2 functions primarily as a cytoplasmic lipogenic enzyme, promoting lipid synthesis and storage [34]. However, cancer cells adapt to the growth conditions of the tumor microenvironment by activating or increasing the expression level of ACSS2 under metabolic stress [35]. Therefore, we believe that ACSS2 acts as an oncogene to affect proliferation and invasion in pancreatic cancer. However, there is still room for research on how ACSS2 ultimately affects tumor proliferation and invasion status by influencing metabolic reprogramming.

Our clinical sample analysis revealed that ACSS2 was more highly expressed in pancreatic cancer tissues than in normal tissues and that its high expression was associated with a poor prognosis in patients with pancreatic cancer. We also validated these results in the TCGA public database ( Figure 1). Our research proved that ACSS2 could promote the proliferation and invasiveness of pancreatic cancer cells ( Figure 2). One of the typical characteristics of pancreatic cancer is that it is surrounded by abundant interstitial tissue, which leads to differences in its metabolic characteristics. Amino acids can be used as a source of energy rather than a raw material for protein synthesis [7]. Whether ACSS2, an important gene for lipid metabolism, is also involved in amino acid metabolism has not been well studied in pancreatic cancer. We subsequently conducted amino acid metabolomics analysis and reported that a decrease in ACSS2 expression leads to an increase in BCAA levels. To explore the molecular mechanism by which ACSS2 affects BCAA metabolism, we conducted RNA-seq and identified BCAT1 among the significantly differentially expressed genes. BCAT1 is present in the cytoplasm and can reversibly catalyze the first step of BCAA catabolism to produce BCKAs. We also found that a decrease in ACSS2 expression did indeed lower the levels of BCKAs. Therefore, we believe that ACSS2 affects the catabolism of BCAAs through BCAT1. However, the internal regulatory mechanism involved is unclear. The regulation of protein molecules by ACSS2 is often achieved by altering the acetylation of its related promoters. We predicted transcription factors in public databases and focused on PPARD. We predict that PPARD will bind to the *BCAT1* promoter region to promote its transcription. We predicted the binding site of the promoter in the JASPAR database, conducted experiments, and found that PPARD can indeed bind to the *BCAT1* promoter region and promote its transcriptional activity ( Figure 4). How ACSS2 affects PPARD is also a question that needs to be explored further. Studies have shown that ACSS2 affects the acetylation of H3K27 and H3K9, and we found through western blot analysis that ACSS2 has a more significant effect on H3K27ac. Therefore, we speculate that ACSS2 may regulate PPARD by affecting H3K27ac in the *PPARD* promoter region. We found that a decrease in ACSS2 can reduce the acetylation level in the *PPARD* promoter region and that the addition of acetate can partially alleviate this phenomenon. ACSS2 utilizes acetate to synthesize acetyl-CoA, which is also one of the steps affecting protein acetylation. Therefore, we demonstrated that ACSS2 is the first step in the regulation of BCAA catabolism, primarily through the H3K27-PPARD-BCAT1 axis.

In the public database and Fudan cohort, we found that BCAT1 is an oncogene in pancreatic cancer. It can catalyze the metabolism of BCAAs to produce BCKAs. BCKAs also participate in the tricarboxylic acid cycle and affect energy metabolism, and they can provide energy for the stress response and biological behavior of tumor cells. Therefore, we believe that the ability of ACSS2 to promote the proliferation and invasiveness of pancreatic cancer cells can be partially reversed by *BCAT1* silencing. We also tested this hypothesis in pancreatic cancer cell lines and organoid models. However, this study also has several limitations. First, we did not validate the proliferation- and invasion-related properties of BCAT1 in reversing ACSS2 expression in a mouse animal model. Second, ACSS2 may also partially regulate the transcription of *BCAT1* by directly affecting the acetylation of the *BCAT1* promoter region, which requires further exploration.

The clinical significance of our research is that ACSS2 can promote the metabolism of BCAAs, suggesting a new approach for treating pancreatic cancer by targeting metabolism. In terms of mechanism, we found that this gene is regulated mainly through the ACSS2-K3K27-PPARD-BCAT1 axis. Moreover, the impact of ACSS2 on invasion and proliferation is partially regulated through BCAT1. These findings can improve the prognosis of patients and provide new ideas for the treatment of pancreatic cancer by targeting molecules of this regulatory axis.
